# Decodification for dyslexia and broader reading difficulties

**DOI:** 10.4102/ajod.v15i0.2087

**Published:** 2026-09-14

**Authors:** Larry E. Onokpite

**Affiliations:** 1 Department of Educational Studies, Special Education Program, College of Education and Human Ecology, The Ohio State University, Columbus Ohio, United States of America

**Keywords:** dyslexia, reading difficulties, deficit, decodification, reading instruction

## Abstract

Dyslexia is often addressed through specialised intervention, yet growing scholarship in literacy and educational psychology also underscores the importance of contextualised and meaningful literacy experiences for students with reading difficulties. In this article, I argue the relevance of Paulo Freire’s concept of decodification for reading instruction for students with dyslexia and broader reading difficulties. Drawing on the literature on dyslexia, background knowledge, culturally relevant text selection and experiences of emergent bilingual learners, I argue that decodified texts may support engagement, meaning-making, vocabulary integration, inferential reasoning and retention. The discussion extends the framework beyond dyslexia to other reading difficulties and illustrates its instructional relevance through three practice-oriented suggestions: recognising the student’s context, talking with the student and evaluating current texts.

**Clinical Implications:**

A pedagogical example further shows how decodification may inform text selection and assessment within existing reading instruction, rather than constitute a new intervention. Overall, in this article, I position decodification as a literature-grounded and practice-relevant framework for rethinking reading instruction in ways that better reflect students’ lived realities.

## Introduction

Dyslexia is a reading disability that arguably requires highly specialised intervention (Shaywitz & Shaywitz 2020). Approximately 15% – 20% of the school population in the United States is estimated to have symptoms of dyslexia (Wagner et al. 2020). There is a growing body of interdisciplinary research on dyslexia and interventions to assist students who have dyslexia. In this article, I am not suggesting a new dyslexia intervention but theoretically inviting practitioners to re-evaluate the materials they use in reading instruction. In the past decade, research across educational psychology and literacy studies has emphasised not only the cognitive dimensions of dyslexia but also the importance of contextualised, meaningful literacy experiences for students with reading difficulties (Johnston & Scanlon 2021:107–128; Smith et al. 2021:214–240; Wolf et al. 2024:303–324).

Although decoding-focused intervention remains essential, reading researchers have shown that reading instruction cannot be separated from the sociocultural, experiential and contextual realities that students bring to the classroom (Muhammad 2020; Tatum 2021). Despite decades of reading instruction research, many students with dyslexia and other reading difficulties still remain disengaged when texts do not reflect their realities. In this article, I make a case for the suitability of Freire’s (1970) theory of decodification, in which he argues that students should be able to see their experiences in the curriculum. Freire argues that decodification can support deeper engagement and meaning-making. To address broader reading challenges highlighted in recent scholarship, my article additionally situates decodification as a framework relevant not only to students with dyslexia but also to students with other reading disabilities and difficulties.

## Decodification, dyslexia and reading difficulties

The concept of decodification forms part of Freire’s (1970) discourse on transformative education. The discourse on transformative education describes a pedagogy that liberates the oppressed and enables them to change their world (context) and the word (learning or education) through critical consciousness – the ability to perceive, understand and critique the sociopolitical and socioeconomic forces that impact the individual and their world (Freire 1970). Freire demonstrates that education must exhibit two integrated features: codification and decodification.

Freire (1970) explains that codification is a recognition and genuine appreciation of the experiences of people in a specific community (context). For him, the priority of the school is to become one with the community to be nurtured and taught by the community. This is important in that it promotes pedagogical advancements that must align with the realities of the community.

The second integrated feature is decodification, which is at the centre of this article. Freire (1970) argues that decodification happens when students can locate their experiences within the school’s pedagogy and curriculum. Here, the teaching and learning materials are contextual to the student – they are relatable (contextually decodable) and filled with students’ experiences (Freire 1970). For Freire, it is easier for a student to achieve a critical appreciation of the schooling process when it is decodified; otherwise, the student is estranged and further alienated from it. Freire (1970) makes this argument:

… ‘decoding’ … requires moving from the part to the whole and then returning to the parts; this in turn requires that the Subject recognize himself in the object (the coded concrete existential situation) and recognize the object as a situation in which he finds himself, together with other Subjects. (p. 105)

Above, Freire emphasises that in the pedagogical process, the student (subject) should be able to see themselves in the teaching and learning materials (object). The teaching and learning materials should support how the student relates to other students and their experiences.

Admittedly, Paulo Freire likely did not theorise for dyslexia or broader reading difficulties, but in this article, I argue that the relevance of his decodification theory holds possibilities for reading instruction and advocacy. For example, in one study (Onokpite 2022) demonstrated how using a decodified text helped in the reading recollection of grade four students with dyslexia at a school in Johannesburg, South Africa. The classroom teacher had remarked about how the students failed to remember the plot of a British novel they read in class. In this study, Onokpite (2022) replaced the British text with an African speculative fiction text and found that the students were able to recollect the latter text. They also commented that it was not like the other text they read before the study commenced. Onokpite (2022) classified the text as decodified from a Freirean perspective because descriptions of towns and villages in the novel reflected the local context and were thus familiar to the participants. This was further evidenced by the visualisations represented in their drawings.

Findings from this study suggest that decodification has potential to be an important element of successful reading instruction that contributes to increased student engagement and learning retention. Furthermore, the principle of decodification extends to broader groups of students with reading difficulties. For these students, familiar and culturally meaningful materials can lower cognitive load, activate background knowledge, support vocabulary integration and facilitate inferential reasoning (Kelly et al. 2023; Sharma & Christ 2017:295–305). For example, emergent bilingual students show improved comprehension when instructional materials reflect their cultural and linguistic worlds (García et al. 2025). This claim aligns with Freire’s argument that relevant, situated and experiential texts improve understanding.

### Decodification in reading instruction

The concept of decodification can be integrated into two areas of reading instruction: (1) in selecting texts for reading instruction; and (2) in assessments. Below I offer three suggestions and one pedagogical application example of how teachers can integrate decodification into their existing instruction in the classroom for students with dyslexia and broader reading difficulties.

One way to understand these suggestions is through Freire and Macedo’s (1987) thesis on *reading the world and the word*. They demonstrated that ‘reading the word’ is encoding or decoding words for meaning-making in relation to a person’s agentive experiences, cultures and knowledge (Rajagopal & Kendrick 2023:94–107). While ‘reading the world’ is to draw upon those experiences, cultures and knowledge to make sense of different systems and one’s place in the dynamics. This thesis builds on the decodification theory earlier postulated by Freire in 1970.

In the following sections, I present a vignette for each of the suggestions to illustrate how each step can be applied in a classroom setting. While the names and scenarios in these descriptions are fictitious, I invite educators to think of the context of the student as the world and the text as the word; this should be an integrative and dialogic process. These suggestions should be treated as iterative engagements and not secluded rigid steps. That is, the recommendation is not to create another form of intervention but rather to offer suggestions for how such interventions can be implemented within existing approaches to reading instruction.

#### Suggestion one: Recognise the student’s context

A classroom is diverse even when all students come from the same community. For example, there could be 12 students in a classroom from a given community. The context of students who come from homes where a cat is considered part of the family will, however, be different from students whose families do not appreciate cats or take them as part of the family. In this case, their understanding of a pet is different. If a teacher were to read a book about pets to these students, their context for understanding the text would differ, depending on the nature of pets being described. That is, the students’ understanding of the nature of pets and thus their understanding of the text would be different because their contextual experiences are different:

- Vignette 1: Uche is a student with dyslexia in Ms. Goodwyn’s classroom in Denver, CO. Ms. Goodwyn has been reading a book about cats with Uche, who is struggling to read the text and remember the content. Ms. Goodwyn is unsure what the problem is since the text is age- and ‘grade-appropriate’. Although Uche is growing up in Denver, her parents are originally from Nigeria, where cats are not primarily regarded as pets. For some religious and cultural reasons, cats are not ‘liked’ as much or considered to be part of the family as is the case in the United States. Uche now lives in Denver with her family, but they still do not own a cat or intend to own one in the foreseeable future. Uche has heard her parents talk about how cats should not be pets.

To understand why Uche is unable to decode this text, Ms. Goodwyn, like other teachers, can take an interest in learning about students’ cultural, community and home perspectives. This information can be gathered from reading books, requesting conversations with parents and going through the biographical data available when first beginning to work with any student. When Ms. Goodwyn better understands Uche’s context, she could then select texts that are more connected to Uche’s experiences. Notably, this vignette is not an anthropological judgement on people from Nigeria but aims to show how students’ contexts could impact how well they read and comprehend what they read. Additionally, while it may not be possible for a teacher to pick a text that represents all students at a time, this awareness could inform the struggles a student might be having, including with text comprehension. An informed teacher will be able to provide better support.

#### Suggestion two: Talk with the student

Arguably, children enjoy discussing what they care about the most; they also enjoy social conversations with relevant adults in their lives (Test, Cunningham & Lee 2010:8–12). Talking with children holds benefits like expanding their vocabularies, increasing critical thinking and igniting their desire or propensity to enjoy reading and be better readers (Test et al. 2010:8–12). For all students, but perhaps especially for students with dyslexia and other reading difficulties, talking about their interests and events in their lives positions them as experts and is a good way for a teacher to gain insight into how to engage students with the decodification process:

- Vignette 2: Jabari is a grade three emergent bilingual student in St. Paul, MN. He recently moved from Tanzania with his family and is learning English at school while continuing to speak Kiswahili at home. His teacher, Mr. Zaccob, notices that Jabari can sound out words accurately but struggles to explain the meaning of the passages he reads. For example, when reading a story about a child and his family on a ski trip, Jabari could pronounce the words but could not retell the story or answer comprehension questions. After speaking with Jabari, the teacher learns that Jabari has yet to have any experience of snow or skiing and is unfamiliar with the contextual references to skiing. To support Jabari’s comprehension, Mr. Zaccob selects other texts that are closer to his experiences, such as texts about athletics, especially short- and long-distance running and soccer, which is common in Tanzania.

This vignette demonstrates how comprehension deficits can be addressed through decodification, especially for emergent bilinguals. Jabari will likely relate to such readings, see his experiences in the text and appreciate the reading process, thus providing a decodified experience for him.

Importantly, the questions a teacher asks during this talk should not begin with reading or be mainly centred around it. The student may not know what they like to read about, especially if they have only read texts that are not interesting to them or are removed from their reality. The teacher should aim to start more generally, to hear what the student enjoys and use that to estimate the books that will connect to their experiences. In sum, the talk that builds the teacher-student relationship should be harnessed to decodify the reading instruction of students with dyslexia.

#### Suggestion three: Evaluate current texts

The next suggestion is to evaluate the current texts used in reading instruction. The teacher could use the first two strategies above to examine if contextually decodable texts are being used for a given student. Some helpful guiding questions include:

- Do these books or texts currently consider the context of the individual student?
- What cultural or familiar experiences of the student guided the text selection?
- Were some agentive experiences of the student engaged before text selection?

Staudt (2009:147–148) asserted that familiarity is one of the characteristics of a good poem for intensive reading practice for students with reading or learning disabilities. Familiar and appealing experiences are important in the reading process for all students and especially those for whom reading is difficult:

- Vignette 3: Ms. Banda is Vincent’s teacher. Vincent is a student with reading difficulties at an elementary school in Cleveland, OH. In the coming weeks, it will be Christmas Day, and Ms. Banda has been reading about the day to Vincent. However, she has observed he has not been quite interested. While talking to a colleague, she was invited to examine if the texts were familiar or consider the context of the student. She found out that Vincent and his family do not celebrate Christmas. Upon realising this context, Ms. Banda decided to read a different text with Vincent, focused more on the holiday season instead of Christmas Day celebrations.

This vignette shows what can happen during the process of evaluating current texts. A teacher might be reading texts that are unfamiliar or alienating to students. In this case, Vincent might not be interested in Christmas Day as his family does not mark it. He might be more interested in reading about the winter season, snow and other holiday festivities. A constant evaluation and re-evaluation of texts for reading instruction will likely provide better success for students.

Table 1 summarises the suggestions I have made so far for easy consideration by educators, emphasising questions that could guide implementation of each recommendation.

**TABLE 1:** Suggestions for decodifying texts.

Steps	Process	Questions to engage the steps
One	Understand the student’s context	What community does this student come from? What are their family values? (religion, ethnicity, etc.) Have they always been in this community? Have I had some time to speak with the family? Have I gone through their biographical information or history form?
Two	Talk with the student	What are my student’s interests? What does she or he enjoy doing for fun? Have I had a ‘talk’ session with this student asking general open-ended questions? Example: ‘What do you like to do during your free time?’ or ‘What do you do on weekends?’ How and when do I create a relaxing environment for this conversation?
Three	Check the current texts	Do these books or texts currently consider the context of the individual student? Are the texts familiar enough?

## Pedagogical application

This section presents how decodification would look in an actual reading lesson plan. The aim is to demonstrate how a teacher might account for the context of each learner within an existing reading lesson. For this step, I chose a grade four Igbo student, Chidiogo, who recently migrated to the United States with her family from Nigeria. Her current reading level is that of a grade three student:

- Lesson goal: Teaching reading fluency
- Learning objectives: Given a familiar text, Chidiogo will read with speech-like intonation and rate and about > 85% accuracy.
- Instructional materials: As Chidiogo has just moved to the United States, her teacher has spoken to her and, through their interaction, realised that Chidiogo is still rooted in her Igbo and Nigerian experiences, plus she enjoys watching or reading about fantasy. In response, the teacher selected, among many options, the book Ikenga by Okorafor (2020) for the reading instruction lessons. Ikenga is a fantasy book set in Igboland and its extended Nigerian culture.
- Other instructional materials: Tape recorder, pens and highlighter.
- Assessment: As the focus of this lesson plan is on reading fluency, the teacher can still use the current assessments available to them, such as the oral reading fluency (ORF) measurement. When the focus is on reading comprehension, questions should be decodified to reflect students’ cultural and experiential frames. For example, questions from a text about the weather (e.g. Kate Messner’s Eyes of the Storm) could be asked to Chidiogo in Celsius and not Fahrenheit, as she is used to the metric system and could still be adapting to the American use of Fahrenheit.

## Final thoughts

Decodification is a theoretical framework with potential to support reading instruction. Freire (1970) makes the case that learning or education benefits students when they locate their experiences in the process. Reading instructions should not be treated as neutral; the texts used should be decodified. That is, students’ contexts should be prioritised.

Decodification as a framework can be extended beyond reading instructions for students with dyslexia to students with general reading difficulties and many general and special education situations. While the approaches proffered in this article could be difficult for a teacher with large classes, they can be incorporated within a multi-tiered system of supports (MTSS), particularly during tier three interventions. Even in large classrooms where individualised reading instruction can be harder to implement at tier one and tier two levels, texts and assessments can be influenced by decodification principles: what texts can be selected and read that are decodable to the larger group in some ways? Decodification is about intentionality and understanding how contextual knowledge can shift reading instruction for students with dyslexia and broader reading difficulties; it is about reading the world and the word. This approach resonates with contemporary calls for culturally and historically responsive literacy (Muhammad 2020) and equity-driven text selection (Sharma & Christ 2017:295–305).
